# MULTISENSORY STIMULATION FOR HOSPITALIZED PATIENTS WITH DEMENTIA: A PILOT STUDY

**DOI:** 10.1093/geroni/igz038.1674

**Published:** 2019-11-08

**Authors:** Sheila L Molony, Christine Waszynski

**Affiliations:** 1 Quinnipiac University, Hamden, Connecticut, United States; 2 Hartford Hospital, Hartford, Connecticut, United States

## Abstract

Introduction: Delirium occurs in up to 50% of hospitalized patients and the risk is higher in persons with dementia. Multi-sensory stimulation environments (MSSE), including trademarked “Snoezelen” rooms, have been effective in achieving positive outcomes in persons with dementia, but there have been no studies in the acute-care setting. Purpose: This pilot study tested the effect of a therapeutic Multi-sensory Stimulation Environment known as “the Hub” in an acute-care hospital. Methods: A sample of 56 patients were randomized to receive usual care or the Therapeutic Hub intervention during hospital days 2-4. Hub activities were multi-sensory and tailored based on preferences and abilities. We will describe techniques to address methodological challenges in the study with acutely ill, cognitively vulnerable participants. We will also present qualitative data describing the experience of participants receiving the Hub intervention, and will present preliminary findings regarding between group-differences in function (Functional Independence Measure), mobility, falls, wellbeing (Warwick-Edinburgh Mental Wellbeing Scale)– and person-environment relationship conceptualized as situational at-homeness (S-EOH). Conclusion: The results of this study will inform future trials on the effects of unique therapeutic environments for hospitalized persons at highest risk for delirium.

